# “You Feel Out of Place”: A PhotoVoice Study of the Impact of Food Insecurity on College Students

**DOI:** 10.1089/heq.2022.0067

**Published:** 2022-08-18

**Authors:** Elise Gahan, Sara Farooqui, Cindy W. Leung

**Affiliations:** ^1^ Department of Nutritional Sciences, University of Michigan School of Public Health, Ann Arbor, Michigan, USA.; ^2^ College of Literature, Science and the Arts, University of Michigan, Ann Arbor, Michigan, USA.

**Keywords:** academic achievement, college students, food insecurity, health disparities, PhotoVoice

## Abstract

**Purpose:**

There is a high prevalence of food insecurity on college campuses in the United States. Quantitative research has shown that experiencing food insecurity during college is associated with adverse academic, health, and social-emotional outcomes. Research is needed to better understand how food insecurity impacts the lived experiences of students. This study aims to understand students' experiences of food insecurity, with a particular focus on their coping strategies and the effects of food insecurity on their health and academic achievement.

**Methods:**

An adaptation of the PhotoVoice method was used to understand how food insecurity affected 10 college students at a large, Midwestern university. Students submitted photos around their experiences of food insecurity and completed an in-depth interview about their photos. The interviews were analyzed for thematic content using an iterative, inductive approach.

**Results:**

Four themes emerged from the results: (1) economic coping mechanisms, (2) behavioral coping mechanisms; (3) alternate ways to acquire food; and (4) psychosocial and academic consequences.

**Conclusion:**

These findings provide context to the negative impacts of food insecurity on students' academic and health outcomes, and add to the growing body of qualitative research that is needed to inform practices and policies to address food insecurity on college campuses.

## Introduction

Food insecurity on college campuses and its associations with sociodemographic factors and academic outcomes of college students are well documented. A 2017 review found that on average, 32.9% of U.S. college students experienced food insecurity, compared to 11.8% in the United States general population that same year.^1,2^ Students coming from a low-income family, first-generation college student, and with a single parent are more likely to experience food insecurity in college.^3^ Furthermore, students with food insecurity are more likely to be African American, work for pay, receive a federal Pell grant or other financial aid, and experience housing insecurity, compared with their food-secure peers.^1,4–7^

Because college is a transition period, adverse outcomes associated with food insecurity share similarities with those of both children and adults, including poorer mental and physical health outcomes. Compared to students with food security, students with food insecurity report higher levels of stress, anxiety, and depression.^1,4,7^ They are also more likely to have poorer quality diets, lower levels of physical activity, more inadequate sleep, and lower self-rated health.^1,8–11^ These disparities can become magnified in adulthood and are correlated with future risks of chronic disease, including diabetes and cardiovascular disease.^12^

The growing body of research on food insecurity on college campuses has raised national awareness of this issue. However, qualitative research of students' lived experiences is largely missing from the narrative. As colleges work to address campus food insecurity, it is important to understand how experiencing food insecurity impacts students' lives to better inform the broader programs and policies to address it. Using an adaptation of the PhotoVoice method, the objective of this study was to understand students' experiences of food insecurity, with a particular focus on their coping strategies and the effects of food insecurity on their health and academic achievement.

## Method

Students from a large Midwestern university were recruited from a larger qualitative research project (*n*=40) focused on exploring students' experiences with food insecurity. The larger qualitative project complemented a campus-wide survey on food insecurity that was administered in the previous semester.^10^ As part of this project, all students who completed an in-depth interview were subsequently asked about their interest in participating in the modified PhotoVoice substudy.

PhotoVoice is a community-based participatory research method where participants take photographs to share their lived experiences related to a study question.^13^ The present study modified the traditional PhotoVoice method by having participants take photos and discuss them with the research team in one-on-one interviews. These modifications were implemented to facilitate students' participation and offer flexibility with students' academic schedules, as well as to preserve anonymity for students who may not feel comfortable using their personal narratives for advocacy.

Out of the initial pool of 40 students, 10 students provided written consent to participate in the PhotoVoice study. The study was described to students as “[using] photography to highlight and understand the impact of food insecurity, or lack of nutritious food, on the health and well-being of students.” Over a 2-week period, students took and submitted 15–30 photos related to the study theme. Afterward, students completed an in-depth interview with the researchers where they selected 10 photos and described them using the SHOWED method: (1) What do you *see* here?, (2) What is really *happening*?, (3) How does this relate to *our* lives?, (4) *Why* does this problem, condition, or strength exist?, and (5) What can you *do* about this problem, condition, or strength?^13^

Follow-up questions were added, when appropriate, to allow students to further describe their photos. Students also reported their food security status over the past 30 days using the six-item Short Form of the USDA Food Security Survey Module.^14^ In brief, students were classified according to USDA guidelines: 0–1 affirmative responses, high or marginal food security; 2–4 affirmative responses, low food security; 5–6 affirmative responses, very low food security. Students were compensated $30 cash for their participation. This study was approved by the University of Michigan Institutional Review Board.

Interviews were transcribed verbatim from audio recordings and checked for accuracy. Data saturation was reached when no new themes were identified from subsequent participants' photos and interviews. Thematic analysis was conducted using an iterative, inductive approach.^15^ The first coder (E.G.) examined all transcripts and photos for emergent themes and developed an initial codebook of themes and subthemes, which were confirmed by two other researchers (S.F. and C.W.L.). A final codebook was confirmed by all three researchers and the first coder (E.G.) applied the coding schema to all transcripts and photos. Questions or discrepancies were resolved by another coder (C.W.L.).

## Results

All 10 study participants were undergraduate students. The mean age was 20.5 years, and 7 students were female. Four students identified as white, two as black, two as Latino, one as Asian, and one as Native American. Five students lived in dorms or other university-owned housing. Four students lived in off-campus housing and one student lived with the parents. Five students had a university dining plan, and five students did not. Five students worked part-time, one student worked full-time, and four students were not currently working. At the time of the study, two students had full/marginal food security, five students had low food security, and three students had very low food security.

Across the photos and interviews, students discussed a variety of experiences and coping mechanisms related to their experience of food insecurity during college. There were four major themes: (1) economic coping mechanisms; (2) behavioral coping mechanisms; (3) alternate ways to acquire food; and (4) psychosocial and academic consequences.

### Economic coping mechanisms

**FIG. 1.**
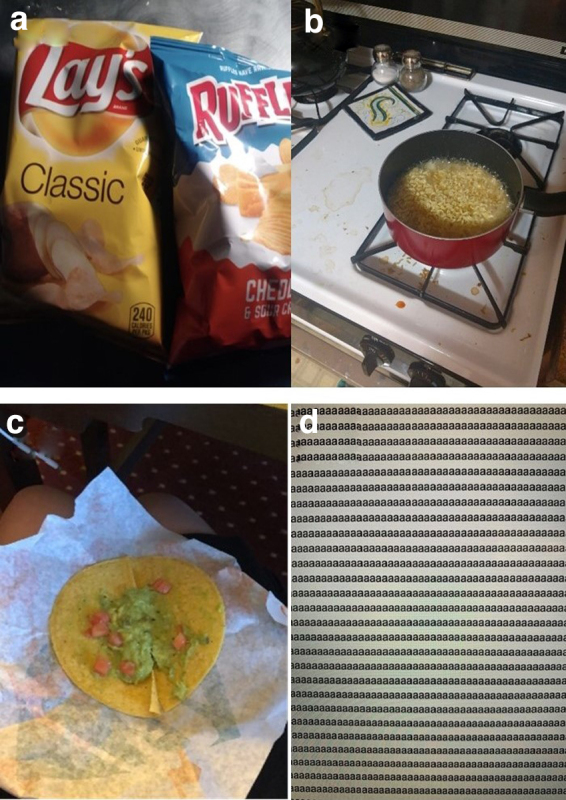
Photos depicting the theme of reliance on low-cost, undesirable, or monotonous foods: **(a)** chips from the vending machine, **(b)** ramen noodles, **(c)** tacos without meat which were the cheapest food available, **(d)** the letter “a” repeated across the screen, which represents the monotony of food available.

Ten students discussed reliance on low-cost, undesirable, or monotonous foods as economic coping mechanisms for food insecurity. Price was often the most significant deciding factor in what foods they would eat. Students prioritized cheaper foods of lower nutritional quality and selected undesirable foods to maximize their food budget. One student took a picture of chips she had for dinner because she could only afford food from the vending machine (Fig. 1a). She explained that she had become desensitized to her experience of food insecurity but realized it was not the norm for many. “I think [my food situation] is pretty pathetic. I kind of laugh about it because I'm so used to it. Maybe for other people it's not as funny.”

Concern over dietary effects of the available food on their physical and mental health was raised in five interviews. Students discussed the tension of the cost of food and its health impacts. Relying on ramen noodles personified this choice for multiple students (Fig. 1b). One female student said “That's ramen and it is definitely not healthy. It's what I had for dinner a couple nights in a row because I couldn't afford groceries.”

Another student commented, “This is one of those meals we get warned about in class. It's high in carbs, just one bag has like 1040 mg of sodium. There's no greens, there's no fiber in it, but it's affordable. It keeps my meal prices way down so then I don't feel bad about eating it.” This student went on to describe how relying on ramen and other nonnutritious food as staples in her diet took a toll on her mental health: “I'm exhausted all the time and I'm sad a lot. I'm ready to cry all the time. It adds to my anxiety. I'm like, ‘Oh, I wonder if people are looking at the fact that I eat really bad food right now.’ ”

Another student felt that her financial situation and chronic shortage of food affected her health (Fig. 1c), “A lot of the foods that I can afford are the ones that aren't good for you which has impacted my health and is also how I became anemic because I didn't get enough iron because I couldn't get meat.”

Monotony was also common among students with food insecurity, with four participants' responses being coded with this theme. One participant shared a photo of the letter “a” repeated across his computer screen to symbolize his experience coping with food insecurity (Fig. 1d). He said, “When it comes down to food, especially in college, you end up eating a lot of the same thing every single day and it gets so monotonous. It's just not the best eating the same exact thing every single day. It gets boring and you get tired of it.”

### Behavioral coping mechanisms

**FIG. 2.**
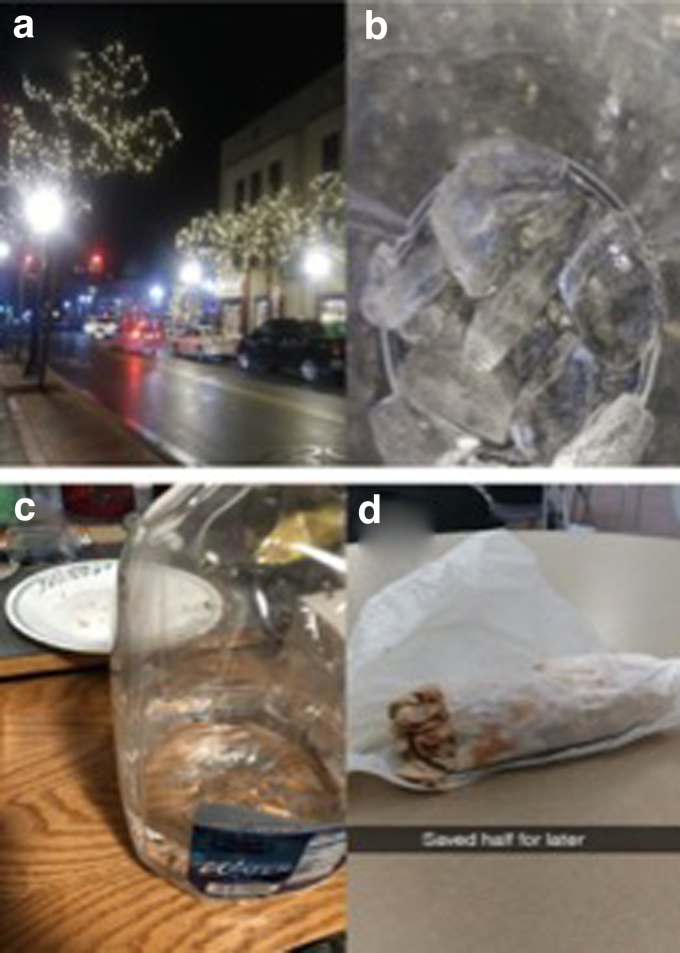
Photos depicting the theme behavioral coping mechanisms for food insecurity: **(a)** downtown lights when the participant had “steps for dinner,” **(b)** ice eaten when hungry in place of food, **(c)** drinking water to reach satiety and to make food last longer, **(d)** a sandwich purchased on campus that was stretched over two meals to compensate for its expense.

Students described behavioral coping mechanisms to manage food insecurity, including ignoring hunger sensations and purposefully not eating enough to feel full to save food. One student discussed three ways, in which she coped with the physiological sensation of hunger. The first way she described was to drink water. She said, “When I don't have food, I will drink a bunch of water. It's so bad.” The second way was to distract herself from eating. She shared a photo showing the surrounding city at night (Fig. 2a). She said, “My original plan was to walk to get food, but I was walking around and thinking I should just save the money. I only had $5, so I walked around to take my mind off of it. I told my friends I had steps for dinner.”

The third way was to eat ice (Fig. 2b): “I eat ice a lot instead of food. I eat ice as a snack, I eat ice as dinner.”

Six students described conserving food by purposefully not eating to satiety. They shared photos of small meals they ate. In Figure 2c, a student described a photo of a water jug in front of her empty plate: “I ate all of the pizza rolls and was not full, but I did not want to eat more or else I would run out. So, I could only drink water.” Other students tried to stretch restaurant or campus food for multiple meals to save money.

In one example, a student forgot her packed lunch at home and purchased a sandwich on campus. She felt guilty about this “unnecessary purchase” and made it last for two meals (Fig. 2d). Another student shared the frequency of eating that she was able to feel fullness, “Maybe about half the time when I eat I feel full. I wish I had more food I could eat, that there was more food available that I could use to make myself feel more full.”

### Alternate ways to acquire food

**FIG. 3.**
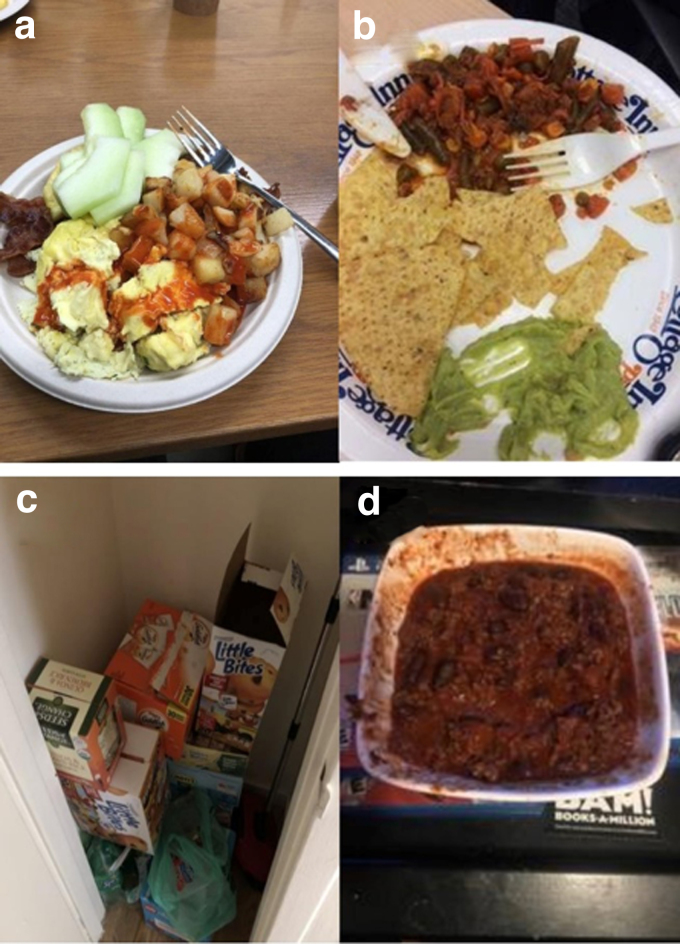
Photos depicting the theme of alternate ways to get food: **(a)** a meal obtained while working, **(b)** free food from a student group meeting, **(c)** nonperishable snacks provided by the student's parents, **(d)** food brought back from a trip home.

Students described alternative ways to acquire food, including gaining employment, joining student groups, relying on their parents, and relying on their peers. One female student, who worked at the campus dining hall, provided a photo of a meal she got after her work shift (Fig. 3a). She described her job as stressful and that a primary reason she took the job was because it would provide free meals: “Obviously, I work to get money, but I also work to get food. No one likes to work in the dining hall. It's not great work but you get fed.”

Figure 3b shows a plate of food obtained from an international potluck sponsored by a student group. This student's motivation for student group involvement was which groups provided free food. Although students appreciated the ability to receive food for free, they expressed concern that foods typically provided at these events, like pizza, were not nutritious.

Five students specifically mentioned relying on their parents or peers to obtain food. One student explained that she couldn't afford a dining plan and relied on food her parents provided for quick meals (Fig. 3c). She explained, “My parents know I do not have money for food when I am at school. Whenever I am at home, they will replace what I have run out of.” Another student described visiting his family to bring extra food to campus (Fig. 3d). He said, “I warn my parents when I'm going to come home and sometimes my mom will cook extra for me. That weekend I not only wanted to see my family, but also I wanted to have food to bring back.”

While some students hide their struggle with food insecurity from their peers, others shared the burden of food insecurity with friends who also struggle to get enough food. One student described how her friends will loan each other money: “If someone does not have money and is hungry, we will pay for it and you Venmo back when you get money.”

### Psychosocial and academic consequences

**FIG. 4.**
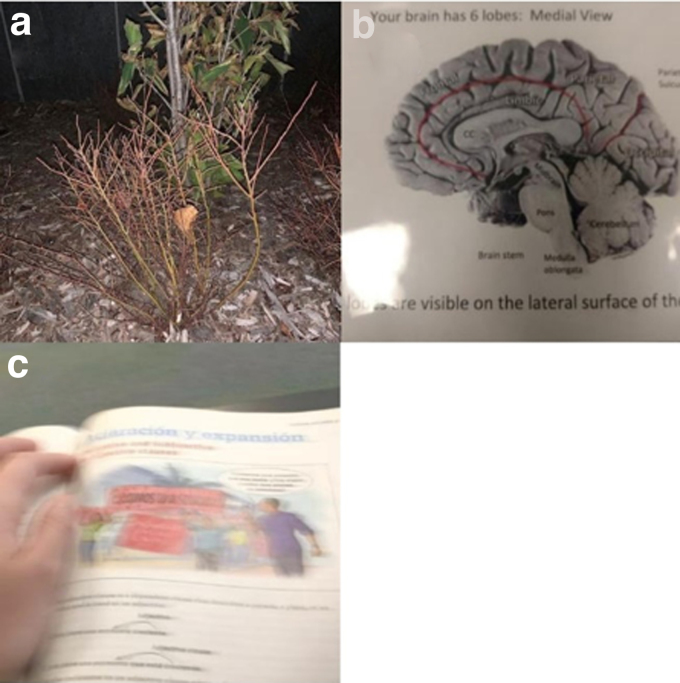
Photos depict the theme psychosocial and academic consequences of food insecurity: **(a)** a bare tree representing that how food insecurity makes the student feel out of place on campus, **(b)** difficulty focusing on class content due to hunger, **(c)** intentionally blurry photo representing how a student's hungry mind feels.

Students experiencing food insecurity regularly found themselves unable to participate in social gatherings, an important aspect of the college experience. One student took a picture of a bush in the winter as a metaphor for his experience (Fig. 4a), “You feel out of place. You don't necessarily belong or fit in… Food is a social thing, and it makes you feel out of place if everyone is with each other and you're not with them.” Another student described having to constantly turn down social invitations because of needing to work to pay for her basic needs. She said, “They'll ask me to hang out and I'll be at work… Because I have to make all this money to support myself, I can't hang out with people.”

Students also described food insecurity negatively impacting their academic performance, both because of the physiological feelings of hunger and the stress of thinking about where to get more food. One student explained, “When I go to the exam and all I ate was chips, all I can think about is ‘oh my god I'm so hungry.’ I can't wait for this exam to be over. Sometimes I eat chips while I'm studying and it's two hours later, then I get hungry, and I get distracted by being hungry. Hunger distracts me from studying.”

Another student described how feeling hungry during class made it harder for her to focus (Fig. 4b): “When you're just thinking about food, it's like, you can't focus for that long and you're like (snaps) come on, pay attention.” Another student intentionally took a blurry photo to demonstrate how her mind feels during her Spanish class (Fig. 4c). “I was sitting in class and thinking about going and getting a snack and I realized that I wasn't at all paying attention to what was happening.”

## Discussion

The objective of this study was to understand students' experiences of food insecurity, with a particular focus on their coping strategies and the effects of food insecurity on their health and academic achievement. Results showed that college students use multiple economic and behavioral coping mechanisms to buffer the harmful consequences of food insecurity, some of which further reinforce negative psychosocial and physical health outcomes.

The first theme showed that students are willing to prioritize cheap, nutrient-poor foods while acknowledging the likely negative health implications. The coping mechanisms students used, including eating smaller portions, buying foods of lower nutritional value, and using distractions were also found in other studies.^9,16–20^ Students discussed their desire to purchase healthier foods, but their lack of money prohibited them from doing so, which contributed to lower diet quality.^11,21^ These findings confirm those of quantitative studies showing that food insecurity among college students is associated with lower fruit intake and higher intake of added sugar.^10,22^ Prior studies have found that interpersonal and environmental factors, including transportation, financial management, and cooking skills can impact students' ability to consume a nutritious diet.^10,23,24^

There have been mixed findings on the role of financial management on food insecurity in college students.^9,25^ Additional research is needed to better understand how these contextual factors influence students' experience of food insecurity and dietary behaviors.

This second theme illustrated that students are acutely aware of their hunger cues, identify it as problematic, and are unable to address it with their available resources. Drinking excessive fluids instead of eating a meal, eating smaller portion sizes, and repeating the same low-cost meals were common coping mechanisms, which mirrored coping mechanisms found in prior research of college students.^17,18,26,27^ Applying these coping mechanism contributed to stress and anxiety as well as inability to focus during classes, demonstrating the reinforcing cycle that food insecurity plays in mental health and academic performance.^27^

The third theme demonstrated the role of protective support systems (e.g., jobs, friends, family, and student groups) in buffering the impacts of food insecurity. Students prioritized availability of food at their place of employment to help them both earn money and not go hungry. Food or money provided by parents or friends was a resource frequently discussed, and students tried to extend how long these resources lasted by not eating to reach satiety. Relying on friends for support differed from two previous studies, which found that students did not want support from family or friends, whereas several students in this study shared how they relied on their parents.^17,27^ However, it aligned with another qualitative finding that support systems provided a protective factor for both mental health and food resources.^17^

The students who relied on peers for support shared that their peers had similar financial situations and understood the stress of not having enough money. As students enter higher education with increasingly diverse backgrounds, further research is needed to understand facilitators to reducing shame and stigma about utilizing resources to combat food insecurity as well as creating opportunities for peers to find social support.

The fourth theme illustrated how food insecurity creates feelings of guilt, anxiety, and loneliness, which affected both students' academic and social life. A systematic review found that food insecurity was associated with lower grade point averages and increased difficulty focusing in class.^1^ Stress, anxiety, and depression were caused by students' perceived inability to take care of themselves independently, engage in social activities, and perform well academically. These feelings left students feeling more isolated and lonely that can exacerbate existing feelings of anxiety and depression.^17,19,27,28^ Food-insecure students are motivated to succeed in college and see their degree as the path to economic mobility.^17^ Educating faculty and staff about the prevalence of food insecurity and resources available to students can improve academic outcomes, decrease stigma, and increase access to resources. Decreasing stigma can also promote students' ability to seek assistance and mitigate the social consequences of food insecurity.^17,19,28^

This study is limited by the modifications made to the PhotoVoice method, which minimized student engagement in the study beyond data collection. In addition, because participation was limited to students who were involved in a prior qualitative study, this study relies on a convenience sample which may not be reflective of the overall student body. This study was also conducted on a large, Midwestern university and may not be generalizable to all higher education environments. Furthermore, the researchers may have their own biases, although this risk was mitigated with all researchers contributing to the analysis.

Although participation has been noted to vary across PhotoVoice studies,^29^ we believe that the themes from the present study will contribute to the broader discussions on university programming, including ways to continue empowering students experiencing food insecurity. For example, at the time of this study, the university where this study was conducted was in the process of establishing a permanent food pantry for the campus community. Results of the present study have served to raise awareness about the prevalence and lived experiences of students experiencing food insecurity and highlighted the need for the university to offer more comprehensive programs to address food and other basic needs insecurities for its diverse student body.

## Conclusions

This study details the lived experiences of food insecurity among a group of college students at a large, Midwestern university. Results showed that despite the variety of economic and behavioral coping strategies used to buffer the negative effects of food insecurity, food insecurity had a clear and adverse impact on their psychosocial and academic outcomes. As food insecurity continues to be recognized as a widespread problem on college campuses, programs and policies can use these findings to center the voices of those experiencing food insecurity to better preserve students' basic needs and ensure their academic success.
